# Stand Up, Students! Decisional Cues Reduce Sedentary Behavior in University Students

**DOI:** 10.3389/fpubh.2019.00230

**Published:** 2019-08-20

**Authors:** Carina Mnich, Philip Bachert, Jule Kunkel, Hagen Wäsche, Rainer Neumann, Claudio R. Nigg

**Affiliations:** Institute of Sports and Sports Science, Karlsruhe Institute of Technology, Karlsruhe, Germany

**Keywords:** sedentary behavior, college students, sitting, standing, physical activity, observation, decisional cues

## Abstract

**Background:** University students are prone to sedentary behavior (SB) which is associated with multiple negative health outcomes. Sit-stand desks may allow for a reduction of SB through standing bouts. To promote standing in university students, decisional cues might be a low-cost approach that can easily be implemented.

**Purpose:** To investigate the effects of decisional cues on students' SB, standing, and active behavior.

**Method:** Over 3 weeks, students were observed in a building on a German university campus, which provides sit-stand-desks in study areas, using an adapted version of the SOPLAY protocol. Baseline data was collected in the first week (T1), before posters and table plaques containing decisional cues were set up in the study areas. Effects were measured in the following 2 weeks (T2 and T3).

**Results:** 2,809 (33% female) students were observed. Sitting decreased from 92.9% [SD = 14.9] to 84.5% [SD = 22.1] from T1 to T3 [*F*_(1, 141)_ = 15.6; *p* < 0.01; η^2^ = 0.10]. Standing increased from 5.6% [SD = 13.5] to 10.9% [SD = 14.4] [*F*_(1, 141)_ = 9.0; *p* < 0.01; η^2^ = 0.06] and being active from 1.5% [SD = 6.9] to 4.5% [SD = 14.8] from T1 to T3 [*F*_(1, 141)_ = 4.2; *p* < 0.05; η^2^ = 0.03). Main effect analyses revealed more students standing in the afternoon compared to morning and lunchtime [*F*_(2, 140)_ = 3.2; *p* < 0.05; η^2^ = 0.04).

**Discussion:** Decisional cues could decrease students' SB and promote standing or being active as alternatives. Future research should use a more rigorous study design. The content of the decisional cues should be explored more and expanded to other health promotion areas on campus.

## Introduction

Sedentary behavior (SB) is referred to as waking behavior in a sitting, lying, or reclined posture with an energy expenditure of ≤1.5 Metabolic Equivalents (METs) (1). SB has been associated with several negative health outcomes, including obesity, cancer, type 2 diabetes, cardiovascular disease, and total mortality along with problems in social behavior and academic achievements (2–5). Deficits in social, behavioral, and academic performance might be due to the distraction of screen-based sedentary behaviors such as TV watching and playing computer games, leading to less time for interactions with peers and studying for school (5). College students are especially prone to SB, as they spend about 10 h a day in SB (6, 7). The SB lasts beyond university, as people with a university degree are likely to be sedentary about 7 h per day the following 10 years (8). Studying is the most prevalent SB among students, followed by TV/video watching and computer time (7, 9). Considering that most sedentary time is spent on studying, and study time should not be decreased due to its impact on academic performance (10, 11), alternatives must be found to substitute SB.

Studying in a standing position may be an option, amongst others. Based on current evidence, 2–4 h of accumulated standing are recommended for predominantly desk-based occupations (12). Standing requires 2 METs (13) and therefore is not classified as SB (1). Standing has been associated with several benefits. Physiologically, higher energy expenditure has been evidenced when standing compared to sitting (14–16). In an Australian sample of adults, for every extra 2 h of standing per day, fasting plasma glucose, total/HDL-cholesterol ratio, triglycerides, and HDL-cholesterol improved, contributing to improved cardio-metabolic health (17). Workers at sit-stand office stations also reported less discomfort, especially in the lower back (18, 19) and in the shoulder/upper extremities (19). Psychologically, the use of sit-stand stations contributed to increased productivity, reduction in work stress, greater worker satisfaction, and quality of life (20), academic engagement and attention (21), and task engagement (22). University students also reported that their attention, participation, focus, and engagement during lectures increase, while boredom, cell phone use, restlessness, and fatigue decrease when standing (23). Based on these results, standing should be promoted as an alternative to SB in university students.

The socio-ecological model provides a useful theoretical framework for this context: It considers human behavior determined by various aspects on different levels, ranging from the political to the individual level (24). On the political level, the Okanagan Charter for Health Promoting Universities is an example for a framework to implement SB-decreasing interventions through “identifying opportunities to study (…) and [simultaneously] support health and well-being in (…) learning environments” and supporting personal development, including competence and life-enhancing skills [(25), p. 7].

While a framework creates opportunities for action on the structural level, such as the built environment, social and psychological factors impact the implementation of the health behavior (26) even if health-supportive structures are provided. Based on these determinants, decisional cues could motivate individuals to engage in healthy behavior (27). Originating from the commercial food industry, decisional cues give immediate information to a consumer about a product during grocery shopping. Thereby, they provide a temporary structure to the consumer, aiming at heuristics and pre-existing, unstructured knowledge for purchase behavior within seconds (28).

Transferring this approach to health promotion, the health behavior is the “product” and making information available when it comes to decide about implementing the unhealthier (sitting) or healthier (standing) behavior could influence individuals' decisions. Aiming at situational decision-making-processes based on heuristics and unstructured knowledge, decisional cues are a complementary approach to theories addressing conscious processes and planned behavior, such as the Theory of Planned Behavior (28) and the Transtheoretical Model (29). Previous studies have shown effects of decisional cues in various health behaviors, including the increase of stair use (30) and healthy food choice (31). However, only two studies could be found that used decisional cues to reduce SB. In a workplace intervention, sit-stand-desks were changed to a default standing height, leading to a 11.3% increase in standing rates (32). In another study, sit-stand workstations were introduced in classrooms and weight-related decisional prompts were placed on top of the desks, leading to 6.6–7.4% more university students standing during lectures (23). While the sit-stand desks of the latter study (14) were introduced in classrooms, the purpose of this present study was to examine the use of decisional cues aimed at reducing SB in university students at sit-stand-desks in open study areas. We hypothesized that the introduction of decisional cues in the form of posters and table plaques at the sit-stand-desks reduce SB. Primary outcomes of interests were the percentage of student sitting and standing, the secondary outcome of interest was active students in the study areas.

## Methods

### Setting and Participants

The mathematics building of a German university was chosen for investigating the effects of decisional cues on student's SB as it has nine student study areas on three levels, each being equipped with sit-stand desks. The sit-stand-desks had been introduced when the building was renovated in 2015 and can be accessed by all students at the campus. However, as these areas are not advertised on campus, it can be assumed that students using them are mostly students attending lectures in the mathematics building. A total of 2,809 students (33% female) were observed in this study.

### Measures

The “System for Observing Play and Leisure Activity in Youth” (SOPLAY) protocol (33) was adapted to fit the study scope. SOPLAY is an observational instrument to obtain data on the activity levels of participants being observed during unstructured leisure activities, based on momentary time sampling (34). Reliability and validity of SOPLAY has been documented (33), indicating objective and consistent measures of students' activity levels. The observation protocol was standardized for this study where the first area observed was always to the right of the stairs and subsequent areas were observed clockwise. Starting with the first level, it was repeated for level two and three. Each area was scanned twice from left to right, once for females and once for males, recording the activity category of each individual observed. To document the impact of our intervention, the SOPLAY activity codes (sedentary, walking, very active) were changed to sedentary, standing, and being active. The definitions for the coding are based on the definitions of the Sedentary Behavior Research Network (35):

1. Sedentary: Any waking behavior with <1.5 METs, characterized by having the main body weight not on the feet, such as sitting, lying, or reclining. Sitting referred to a position with an upright back and the body weight mainly on one's buttocks rather than on one's feet. Lying referred to a horizontal position and reclining to a position between sitting and lying (35).
2. Standing: A stationary behavior while being in an upright posture with the main body weight on the feet (35).
3. Active: Any activity with more energy expenditure than sedentary or standing, such as walking.

External characteristics were recorded for each observation to document the condition of the environment, including weather and temperature outside, availability and usability of sit-stand-desks, accessibility of the building, and if there were special events. An additional comments field was provided on the observation sheet to allow the documentation of any relevant environmental characteristics that have not been considered above. Six observers were trained by a certified SOPLAY instructor. The training included an explanation of the concepts and methods, and on-site practice-coding and documenting activity levels in the study areas. After the training was completed, all observers observed the same study area independently from each other, documenting gender and activity levels, and then compared their results with the SOPLAY instructor, showing an interobserver agreement of 98%.

### Procedures

Written consent of the mathematics' department chair was obtained prior to observation begin. The study was conducted in accordance with the Declaration of Helsinki and the ethical guidelines of the KIT. The observations were anonymous as no identifying information was collected, therefore exempting ethics approval. The 3-week-study was conducted during the winter semester 2018/2019. Observations were taken on Tuesdays, Wednesdays, and Thursdays of each week. Observation times were selected to fit the transition times between lectures in the mathematics building and to cover morning, lunchtime, and afternoon during each day (see Table 1). The time at 1:20 p.m. was chosen consistently, as the university takes a general lunchbreak between 1 and 2 p.m. which students use for studying, doing homework, and having lunch.

**Table 1:** Observation times and weekdays for each data collection week.

**Coding**	**Tuesdays**	**Wednesdays**	**Thursdays**
Morning	11:45 a.m.	10:00 a.m.	9:00 a.m.
Lunchtime	1:20 p.m.	1:20 p.m.	1:20 p.m.
Afternoon	2:30 p.m.	5:30 p.m.	4:15 p.m.

At total of 243 observation scans for men and women, respectively (total *n* = 486), were obtained during the 3 weeks. Pretest-data was collected in the first week (T1). Observers had a set observation order for the study areas. One set of observations, including scans of study areas 1–9 for males and females, was conducted by a single observer. Each area was scanned from left to the right, first for women, then for men. The number of participants sitting, standing, or being active was noted on a standardized observation sheet, before moving on to the next study area. The study areas were surrounded by glass walls so that the observers were standing outside of the study area while conducting the scans and documenting the activity coding. The scans and coding included all people present in the study areas even if they were not at a table. None of the students or employees except for the department chair of the mathematics building was informed about the study purpose. In case of questions, all observers were instructed to answer that they were counting the number of students in the study areas.

**Figure 1:**
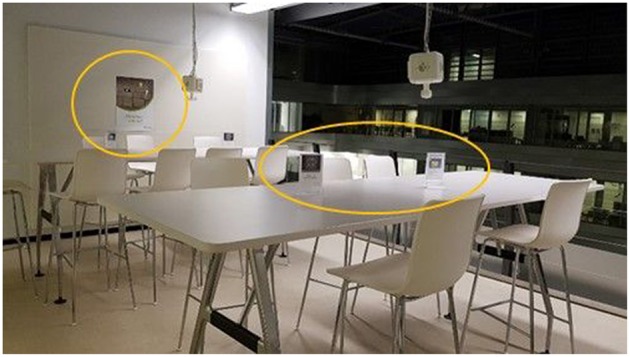
Sit-stand-desks in a study area with intervention poster and table plaques set up.

At the beginning of the second week, one poster and five table plaques were put up in each observation area to motivate students to stand (see Figure 1). The poster was hung at a poster display board, being the only poster that was displayed. Two table plaques were set up on long tables and one table plaque was set up on the small round tables (see Figure 1).

The poster and table plaque content was based on behavior change techniques, using motivational prompts/cues and providing information on the link between health, behavior, and consequences (36, 37). Posters had different pictures and phrases across study areas and were in German (in English: “You better stand up to get fit through your life!”; “Standing up means studying easier!”; “Be smart, stand up!”) with each design represented once at each level. The table plaques all contained the same pictures and information and were in both German and English. On the front it said “Are you still sitting or already standing?,” while the back read “Easier learning through standing,” supplemented by three bullet points giving the information that standing improves attention and focus, memory, and energy expenditure. The information on energy expenditure was used as weight management is a motivational aspect for university students to be active (38), while the information on improved attention and focus was used assuming that students coming to an open study area might prioritize this information.

The same observation procedure as in week one was conducted in weeks two (T2) and three (T3). In the second intervention week (T3), students seemed to have interacted with the table plaques. On the first floor, all motivational signs had been exchanged with creative writings and quotations of students of German poets and modern artists while on the second floor, all table plaques had disappeared. On the third floor, all table plaques were still present and still with the original motivational signs. The table plaques were not replaced considering the intention-to-treat principle. Posters had remained present in all study areas on all floors throughout the intervention weeks and were left up after data collection was over. The remaining table plaques were removed after T3 measurement.

### Analysis

The study hypothesis was analyzed using a 2 (sex) × 3 (time point) and 3 (day part) × 3 (time point) repeated measures ANOVA in SPSS 25. As no activity code could be noted when no students were present during a scan, when zeros were recorded for all 3 activity categories for one scan, that scan was treated as missing data (39). The statistical significance level was set a priori to α < 0.05.

## Results

At T1, 900 students were observed (5.6 in average per scan), 977 students at T2 (6.0 in average per scan), and 932 students at T3 (5.8 in average per scan). During 23 scans, no students were present in the corners (missing data = 0.05 %).

**Figure 2:**
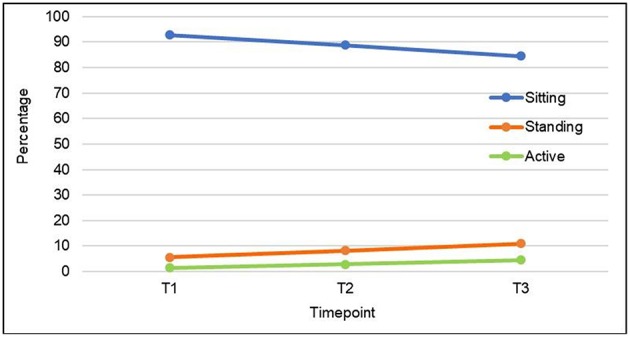
Sitting, standing, and being active over 3 weeks.

There were no interactions of time with gender nor main effects for gender (*p* > 0.05). The time main effects were: Sitting decreased from 92.9% [SD = 14.9] to 84.5% [SD = 22.1] from T1 to T3 [*F*_(1, 141)_ = 15.6; *p* < 0.01; η^2^ = 0.10; see Figure 2]. Standing increased from 5.6% [SD = 13.5] to 10.9% [SD = 14.4] from T1 to T3 [*F*_(1, 141)_ = 9.0; *p* < 0.01; η^2^ = 0.06). Being active also increased from 1.5% [SD = 6.9] to 4.5% [SD = 14.8] from T1 to T3 [*F*_(1, 141)_ = 4.2; *p* < 0.05; η^2^ = 0.03].

**Figure 3:**
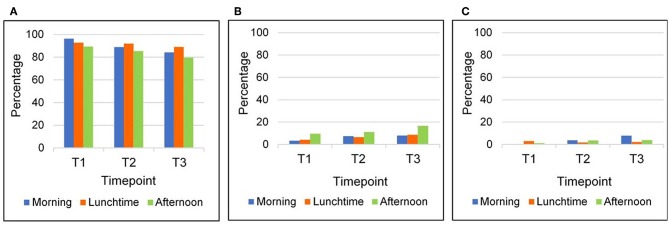
Sitting **(A)**, standing **(B)**, and being active **(C)** over time and during different times of day.

An interaction could be found for times of the day for the active coding [*F*_(2, 140)_ = 7.0; *p* < 0.01; η^2^ = 0.09], with a stronger increase in being active in the morning than in the other parts of the day (see Figure 3). No other interactions were significant (*p* > 0.05). Main effect analyses revealed more students standing in the afternoon compared to morning and lunchtime [*F*_(2, 140)_ = 3.2; *p* < 0.05; η^2^ = 0.04].

Considering the context variables, all sit-stand-desks were available and usable, and the building was accessible through the 3 weeks. Weather and temperature were similar across the 3 weeks and no special events had occurred. Due to the disappearing and exchanging of the motivational signs in the table plaques, we ran a *post-hoc* analysis for effects of the different floors. Neither interaction nor main effects with floors could be found (*p* > 0.05).

## Discussion

The primary aim of this study was to test the hypothesis that decisional cues such as posters and table plaques decrease SB in university students and increase standing rates. The secondary outcome of interest was to see if the active rates also change. Both hypotheses were confirmed. Sitting behavior decreased by an average of 8.4%, standing rates increased by 5.3% in average, and, referring to the secondary outcome, being active increased by 3%. Putting this in the context of impact [= reach X efficacy; (40)], of the 2,809 students observed, 225 students fewer were sitting after the decisional cues had been introduced. Considering that this intervention could be easily implemented in other places with larger student populations (e.g., the library or the whole campus), it could reach thousands of young adults, with relatively low-costs. Thus, this intervention has the potential of a significant impact on the college student population.

The results are similar to previous intervention studies to promote standing (23, 32). Jerome et al. found a 6.6–7.4% increase in standing during lectures when providing sit-stand-desks and weight-related decisional cues. However, the comparison group in this study was a lecture with sit-only desks, so that it is not clear if the increase of the standing rate was only due to the introduction of the sit-stand-desks or the combination with decisional prompts (23). In two studies reported in one paper with primary school children, the introduction of sit-stand-desks led to a 9–10% decrease in sitting behavior, but only in one study the decrease was different from the control group. No decisional cues were provided, but the teachers were informed about strategies on how to reduce SB time during class (41). Considering the class setting, changes in the class structure might also be promising for university courses. In a workplace intervention, default nudging was used by setting adjustable sit-stand-desks to a standing height, leading to an 11.3% increase in standing, which is about twice as much as in the current study. A reason for this difference might be the default nudge in the workplace intervention as the decision to change the height has already been made for study participants, thereby making it easier to decide for the non-SB (42). Transferring default nudging to the study area setting, an option could be taking away the chairs of some desks. This might nudge people from the automatic behavior of sitting down when a chair is available to an automatic standing behavior as they would otherwise have to find and relocate a chair in which to sit (43). Another 19-weeks workplace intervention promoting use of sit-stand-desks also sent out daily e-mail reminders through the first 2 weeks, leading to a 10% decrease in SB and 15% increase in standing (44). In another workplace intervention, participants were provided sit-stand-desks and received weekly emails reminding them of their goal to reduce their sitting time by 50%, leading to a 21% decrease of SB (45). The larger SB decrease in this workplace intervention compared to the present study might be due to the combination of weekly e-mail reminders as well as using goal setting for behavior change (37). Transferring these results to the present study, future motivational cues could also be framed as challenges such as “Can you stand every study hour for 10 min?,” thereby suggesting goals for students. The lack of difference between T1 and T2 in the present study might have been due to the short time period between setting up the posters and table plaques and measurement. Decisional cues may need some time to show an effect. The data also shows that the intervention was more effective for the active coding in the morning than in other parts of the day. One reason could be the context of the message content. Students coming to study areas in the morning probably go there for an effective study time, which fits the message content that indicates better concentration and focus if not being sedentary. None of the cues were tailored to the context of having lunch. As can be seen in the graphs, SB was highest during lunchtime at both intervention measurements. A possible solution would be to use rotating decisional cues (for example on a screen), that fit the context of the time of the day. Looking at the day times, more students were standing in the afternoon compared to morning and lunchtime. Standing in the afternoon may be more attractive after having had classes in a sitting position.

Considering the socio-ecological model as a base, the study results show that interventions targeting the structural level, such as the built environment, should be accompanied by motivational interventions on the individual level to target behavior change on multiple levels. Utilizing such a system perspective enables a comprehensive and effective approach to address health problems. This study does not come without limitations. Data were obtained through momentary time sampling, so that position changes were not documented. Additionally, no control group was used limiting causal interpretations. Further, the intervention was time-limited, not allowing the investigation of long-terms effects. These limitations notwithstanding, the decisional cues seemed to decrease SB in students. As university education is a predictor of high SB, showing and establishing alternatives to SB may contribute to lower SB levels in later life (8). Decisional cues are a potential low-cost intervention to promote a decrease in SB of students on campus. Prospective studies examining the effect of decisional cues on SB should apply a more rigorous research design, including a control group and longer follow-up periods to investigate the long-term effects of the cues. Interventions should also consider setting up cues that cannot be or are less likely to be taken away or changed by students.

Future research should explore variations in decisional cues for the same health behavior, exploring decisional cues to reduce SB in other campus buildings/settings as well as matching the setting and the context of the target group. Applied to other areas on campus, decisional cues may be an effective way to improve SBs of students on campus with relatively low effort and low costs. Additionally, it may be prudent to apply decisional cues to other health behaviors and choices on university campus in future studies, such as standing during lectures, food choices in the dining hall and at snack and drink machines, as well as break behavior (e.g., decreasing alcohol and substance use) among university students.

## Data Availability

The datasets generated for this study are available on request to the corresponding authors.

## Ethics Statement

Written consent of the mathematics' department chair was obtained prior to observation begin. The study was conducted in accordance with the Declaration of Helsinki and the ethical guidelines of the KIT. The observations were anonymous as no identifying information was collected, therefore exempting ethics approval.

## Author Contributions

CM conceptualized and designed the study, guided data collection, analyzed the data, and drafted the manuscript. PB regularly communicated with the first author and provided critical feedback at all stages. JK as principle investigator wrote the grant with RN and PB, supported by HW, and supervised the project. CN provided guidance on the study design, data collection, and analysis. RN, HW, JK, and CN provided valuable input after the first draft of the manuscript. All authors contributed to manuscript revision, read, and approved the submitted version.

### Conflict of Interest Statement

The authors declare that the research was conducted in the absence of any commercial or financial relationships that could be construed as a potential conflict of interest.
